# The Effect of Martial Arts Training on Cognitive and Psychological Functions in At-Risk Youths

**DOI:** 10.3389/fped.2021.707047

**Published:** 2021-10-22

**Authors:** Anna Harwood-Gross, Bar Lambez, Ruth Feldman, Orna Zagoory-Sharon, Yuri Rassovsky

**Affiliations:** ^1^Department of Psychology and Gonda Multidisciplinary Brain Research Center, Bar-Ilan University, Ramat Gan, Israel; ^2^Center for Developmental, Social, and Relationship Neuroscience, Interdisciplinary Center, Herzliya, Israel; ^3^Department of Psychiatry and Biobehavioral Sciences, Semel Institute for Neuroscience and Human Behavior, University of California, Los Angeles, Los Angeles, CA, United States

**Keywords:** martial arts, oxytocin, cortisol, youth delinquency, executive functions

## Abstract

The current study assessed whether an extended program of martial arts training was a viable intervention for at-risk youths in improving cognitive and psychological functions. Adolescent boys attending specialized education facilities for at-risk youths took part in regular sport lessons or martial arts practice twice a week for 6 months. Hormonal reactivity was assessed during initial training, and measures of psychological (aggression, self-esteem) and cognitive (inhibition, flexibility, speed of processing, and attention) functions were assessed before and immediately following the intervention. Participants in the martial arts training demonstrated significant improvement in the domains of inhibition and shifting and speed of processing. Additionally, initial hormonal reactivity (oxytocin and cortisol) to the intervention predicted significant post-intervention change on several measures of cognitive and psychological functioning. Specifically, oxytocin reactivity predicted improvement in processing speed, as well as reduction of aggression, whereas cortisol reactivity predicted increases in self-esteem. This pioneering, ecologically valid study demonstrates the initial efficacy of this enjoyable, readily available, group intervention for at-risk boys and suggests potential mechanisms that may mediate the process of change.

## Introduction

A substantial body of literature suggests that a failure to complete education at a normative school setting is associated with important problematic behaviors in youths. Commonly found at the fringes of society, youths unable to function in normative educational settings are often characterized by high levels of externalizing behaviors, which lead to disruption in normal classroom settings and eventually exclusion to non-traditional educational settings (1). As a consequence, youths struggling to complete education at a normative school have been colloquially defined as at-risk (2). They are considered at risk of completely disconnecting from society and family structures and in turn at-risk of future criminality and delinquency (3).

Originally studied as an intervention for at-risk youth in 1986, martial arts were suggested to reduce delinquent personality tendencies (4). In the decades that followed, they have been suggested as an intervention to reduce aggression and are commonly offered with socially vulnerable youth and as supplementary youth programming (5). It has been theorized that martial arts reduce aggressive tendencies by enabling the participant to channel such energies into productive and self-enhancing activities (6), and that the development of self-control coupled with an enhanced awareness of self-boundaries may furthermore contribute to these observed psychological benefits (7). Indeed, common to all the recently reviewed studies demonstrating reductions in externalizing behaviors following martial arts practice were the capitalization of training on complex, repetitive movements, self-controlled behavior, and interpersonal respect (8). Although these are all plausible explanations for the suggestion that martial arts practice reduces aggression, they have little empirical backing, and no studies have evaluated potential pathways of change. Just as the enhanced boundaries, respect, and self-awareness have been linked to reductions in aggression, so too have they been associated with the observed increases in self-esteem (9). Martial arts have furthermore been proposed to be beneficial for character building, and self-esteem enhancing programs are common, with little research backing such claims (10). Despite their widespread use, and even the suggestion of inclusion in the criminal justice service (11), there is scant evidence on their efficacy with at-risk youths.

Forming the foundation for the current study, a recent meta-analysis demonstrated the potential for martial arts to reduce aggressive behaviors (8). Despite the promising findings, only twelve studies were relevant for inclusion, and just one studied aggression in at-risk youths (12). While aggressive behaviors are the most widely studied effects of martial arts, recent studies suggest that martial arts may also improve cognitive function, primarily executive functions (13, 14), which have been repeatedly associated with delinquency, criminality, and at-risk youths (15).

It has been hypothesized that the improvement in cognitive functions following martial arts may be due to the repeated usage and targeting of specific executive functions, primarily self-control and inhibition (16). Combining this specific targeting with aerobic activity may also increase the efficacy of executive function improvement (17). It appears that in populations with ADHD, sports interventions, which directly challenge executive functions, induce the greatest cognitive improvement, more so than even ADHD-specific psychological interventions (18). Martial arts require participants to monitor their behavior, initiate attacking or defensive moves while preventing themselves and their opponent from injury and consistently modify their moves based on the partner's response or attack. Unfortunately, the effect of martial arts on executive functions has not been evaluated in at-risk populations.

The current study was part of a wider study which addressed the lack of research into the mechanisms of change during martial arts practice. Mechanisms of change studied included the effect of martial arts practice on hormone production throughout a practice session. Given the dyadic and bonding nature of martial arts, it was suggested that the neuropeptide oxytocin (OT), which is associated with social affiliative responses (19) may respond to martial arts practice. Various physical activities have induced OT responses (20, 21), but as martial arts combine close social activities, interpersonal contact, and physical activity, it was suggested that they may be particularly conducive to an OT response. Additionally, the glucocorticoid hormone cortisol (CT), which is particularly reactive to stress, was also predicted to increase throughout the practice session, although studies measuring CT reported conflicting responses to physical activity, in general, and martial arts, in particular (22).

The hormonal changes during martial arts practice were studied in low-risk, martial arts hobbyists (23) and at-risk youths (24). Salivary OT was found to increase regardless of martial arts experience. This increase in OT was specifically relevant to the at-risk youth sample, as the increase persisted following cessation of practice, leading the authors to question whether martial arts may have differential effects for at-risk and low-risk youths (24).

Following these findings, the current exploratory study was designed to further explore the effects of martial arts on cognitive and psychological functions in at-risk youths. Specifically, we sought to evaluate whether martial arts training would have an effect on measures typically associated with executive functions, including behavioral inhibition and set shifting, speed of processing, sustained attention, and flexibility and problem solving, as well as on self-report psychological measures of aggression and self-esteem. Furthermore, given the aforementioned findings on the impact of martial arts on salivary OT (24), we also examined whether hormonal changes (OT and CT) witnessed following an early training session, would influence the outcome of long-term martial arts practice. To this end, at-risk youths who provided saliva samples during an initial martial arts session were tested following 6-months of martial arts training on cognitive and psychological outcome measures, thus enabling to examine the effect of the hormonal change on predicted outcomes from martial arts practice. Given the predominantly positive effects of martial arts on behavioral and cognitive outcomes it was predicted that martial arts would improve aggression, cognitive and self-esteem measures, even in at-risk youths, but that these may be impacted by initial hormonal response.

## Methods

### Participants

Forty-nine boys were recruited from two schools for at-risk youths, located in low socioeconomic areas, including San Martin in Jerusalem and Ramle. Initial contact was made with the school principal, who, after authorizing the project, facilitated the communication with the head teachers. The head teachers of each classroom coordinated presentations of the study to students by the researchers during class and were instrumental in integrating the martial arts program into the general curriculum (after receiving approvals from the relevant ethics committees and parental consent, as described below). Teachers were not involved in any of the intervention procedures or data collection. All participants had specific educational needs (ranging from disruptive behaviors to non-attendance at previous educational establishments due to behavioral issues) but were all in regular high-school matriculation classes with additional classes for students with significant learning disabilities. These schools are considered as “last-resort” attempt to keep enrolled students in the general education system.

Only boys were included in the study to avoid potential selection bias, given the substantial over-representation of boys, reflecting the nature of these educational systems in dealing with severe externalizing behavioral problems. The boys ranged in age from 14 to 17.7 years (*M* = 15.6, *SD* = 0.81). Six participants had a formal ADHD diagnosis and were prescribed Ritalin, and nine participants at least one parent attended university (For demographic characteristics see Table 1).

**Table 1:** Participant characteristics.

	**Martial Arts**	**Controls**	**Statistic**	***p***
	**(*n* = 24)**	**(*n* = 25)**		
Age	15.8 (0.78)	15.4 (0.78)	*t* = −1.97	0.06
*M* (*SD)*				
Extracurricular physical activity	4.05 (1.36)	3.41 (1.33)	*t* = −1.54	0.13
*M* (*SD)*				
ADHD diagnosis	9.5%	21.1%	*χ^2^* = 1.04	0.31
At least one parent attended university	14.3%	31.6%	*χ^2^* = 1.71	0.19

### Cognitive and Psychological Measures

Data were collected at two time points: at baseline and post-training. Baseline data were collected during the first weeks of the school year, 1–4 weeks prior to the intervention, using iPads pre-installed with the *Cambridge Neuropsychological Test Automated Battery* (CANTAB; (25) and questionnaires directly inputted into the Qualtrics research and production software. Post-training data were collected in the same manner during the 2 weeks following the intervention. Data were anonymized by assigning participants random unique codes, and only the lead researcher had access to full coding information. All research assistants were trained in the testing procedures by the lead researcher.

#### CANTAB Cognitive Assessment Software

Participants were tested using the CANTAB (25) executive function battery, which included the Multitasking Test (MTT; testing multitasking and stroop-like interference of incongruent information), Rapid Visual Information Processing (RVP; a measure of processing speed), and One Touch Stockings of Cambridge (OTS; similar to the popular Tower of Hanoi test). The following primary key measures of executive functions were included in the analyses: inhibition and shifting rate (the difference between the median latency of response during assessed trials in which multiple rules are used vs. assessed trials in which only a single rule is used on the MTT), speed of processing (median response latency on the RVP), sustained attention (sensitivity to target sequence on the RVP), and flexibility and problem solving (problems solved on the OTS at first attempt). Mean change scores were calculated by subtracting time 2 scores from time 1 scores.

#### Rosenberg Self Esteem Scale

The RSES (26) is one of the most widely used measure of self-esteem (27) and has been used frequently with adolescents, often showing correlation with other measures of life satisfaction during and following the adolescent period (28). It has demonstrated high internal consistency (Cronbach α across cross-cultural studies averages 0.81) (26). The scale consists of 10 items, half of which are written in the positive and half in the negative (with scoring reversed). Scoring is between 0 and 30, with a normal range being between 15 and 25. All items are answered using a 4-point Likert scale format ranging from strongly agree to strongly disagree. Higher scores indicated higher self-esteem, and a total raw score is used for analyses.

#### The Aggression Scale

This is a self-report questionnaire, (29) with very good internal consistency (Cronbach α averages 0.87). Participants are asked to rate how many times during the past week (0–6+) have they performed 11 types of aggressive behaviors, such as “I pushed other children” and “I threatened to hit or harm someone.” The total raw score is used for analyses. The questionnaire was validated with adolescents and showed good construct validity with other measures of aggression including behavior records (29).

### Saliva Sampling and Analysis

At first week of training, three saliva samples were collected: (1) baseline, (2) after the peak training intensity, (3) following a cool-down period [see (23)]. Saliva was collected by participants drooling into a clean 5 ml tube. Participants were told they can drink water immediately after each saliva transmission but avoid drinking until the next saliva sampling. Samples were stored at −20°C.

The concentration of OT was determined by Cayman-OT (Cayman Chemicals, Ann Arbor, Michigan, USA) ELISA kit (enzyme-linked immunosorbent assay). The kits were used for analyzing hormones in saliva. In order to prepare the sample for measurements, samples underwent three freeze-thaw cycles, with freeze at −80°C and thaw at 4°C to precipitate the mucus. The tubes were subsequently centrifuged at 1,500 g (4,000 rpm) for 30 min. The supernatant was transferred into a clean tube and stored at −20°C until assayed. Concentration of OT in these samples was determined in duplicates according to the manufacturer's kit instructions. The inter-assay coefficients of samples and controls were <18.7%, which is in the range reported by the manufacturer.

The concentration of CT was also determined by using a commercial ELISA kit (Salimetrics, USA). Measurements were performed according to the kit's instructions. In addition to the manufacturer's low and high controls (1,060 + 270, 9,700 + 2,430 pg/ml), three in-house controls were included in each plate (250, 880, 1,330 pg/ml) to correlate between plates measured in different periods. Concentration of CT was calculated according to relevant standard curves. The intra-assay coefficient of variance (CV %) of manufacturer and in-house controls is 7.54%. The inter-assay CV of samples is <16.11%.

### Procedure

Participants were divided according to their school timetable by the head teachers into a martial arts training group (*N* = 24) and a control group (*N* = 25). Randomization was not possible, as only those who had free slots in their highly structured timetable were able to be included in the intervention group. The intervention was thus added to the school timetable in addition to other vocational classes (such as maintenance and carpentry) and those with free slots at the time of the martial arts classes were included in the intervention group. As can be seen in Table 1, no significant differences between the control and experimental groups were found for age, ADHD diagnosis, parental education, and rate of extracurricular physical activity (all *p*'s >0.05). Those in the martial arts training group received two 50-min martial arts classes per week included in their school schedule for a 6-month duration (November through April). The control group received the same number of standard PE classes (football, gym, and mixed sports). The standard PE classes were a mixture of sports and varied throughout the year. These classes were matched on time to the martial arts training but were not structured to target specific skills. The martial arts classes included standard elements of Karate, Judo and, Jujitsu and were taught by instructors experienced with at-risk youths in sports rooms equipped with mats to become *dojo*s (practice spaces). The practice was considered traditional, soft-contact martial arts (30). All martial art forms were conducted in a traditional manner but were adapted to participants with special behavioral needs by maintaining all interactions non-competitive and easy-to-contact. All sessions included warm up, movement and technique practice, *randori* (high-intensity, free-style friendly tournament), and cool-down periods. Participants in the control group continued with their standard educational timetable, which included weekly physical education classes of varied sports.

Research assistants (undergraduate and graduate psychology students) were trained on the administration of all measures by the study primary investigator and were blind to group allocation when performing baseline and post-intervention assessments. Students were tested individually by the research assistants who accompanied each participant into an empty classroom and remained with them during testing, in order to answer questions and reduce the chance of random or negligent responding by the participants. All participants provided parental informed consent for their participation. The research was approved by the Institutional Review Board at Bar-Ilan University, the Israel Ministry of Education Ethics committee, and the Helsinki Ethics committee of Hadassah Hospital in Jerusalem, as required by the Ministry of Health.

### Statistical Analysis

Analyses were conducted using *jamovi* statistical software (31). Due to group differences at baseline, change scores (differences between baseline and post-training) were calculated, and independent-samples *t*-tests were performed to analyze group differences. Hormonal reactivity (OT and CT) was calculated by subtracting baseline from peak-training values, as well as by using standard formulas (32) to compute the areas under the curve with respect to ground (AUC_G_) and with respect to increase (AUC_I_). Linear regression analyses were then performed to examine whether hormonal reactivity to the intervention predicted change on outcome measures. When cognitive measures did not contribute to the regression they were removed from final analysis. During the second week of training, class schedule was modified, resulting in scheduling conflicts with vocational courses. As a result, 6 students were reallocated between the experimental and control groups (2 in experimental and 4 in control), and 2 additional students in the control group had to stop training altogether. For 2 additional participants in the experimental group, there was a discrepancy between taking Ritalin during the baseline and post-training testing, excluding their cognitive data from the final analyses. The final sample for analyses consisted of 20 participants in the martial arts training group and 19 in the control group.

## Results

**Figure 1:**
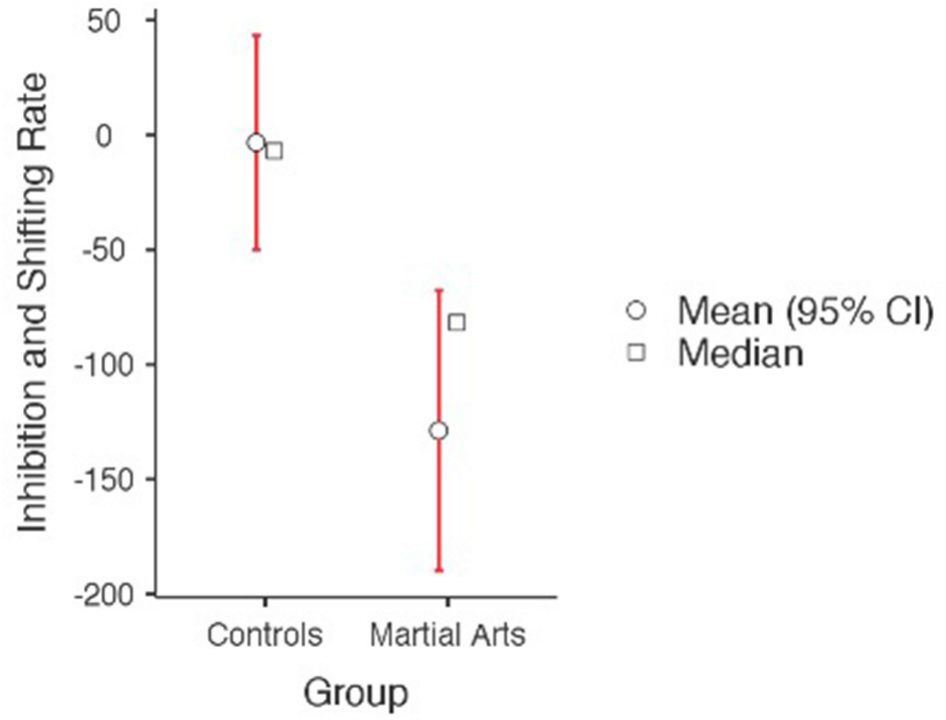
Performance on CANTAB multitasking of inhibition and shifting rate (higher scores reflect poorer performance). A significant group difference was found, *p* = 0.003.

**Figure 2:**
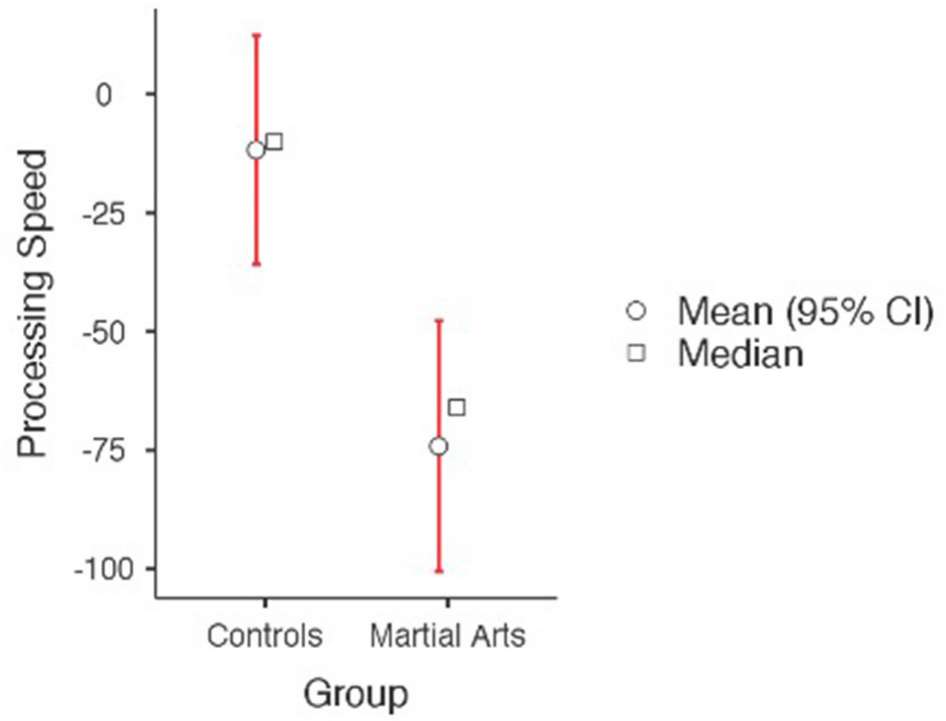
Performance on CANTAB speed of processing task (higher scores reflect poorer performance). A significant group difference was found, *p* = 0.002.

Independent-samples *t*-tests were used to compare cognitive and psychological change scores (differences between baseline and post-training) between the martial arts and control groups. There were no significant differences between the martial arts experimental group and the control group on the key study variable of aggression or self-esteem. As can be seen in Table 2, compared to controls, following the 6-months martial arts training, the martial arts experimental group demonstrated a greater improvement in multitasking during high cognitive load of inhibition and shifting rate, indicating an improvement in managing multiple sources of information and cognitive flexibility (Figure 1). The martial arts experimental group also demonstrated a significant improvement in processing speed, indicating a significantly higher increase in the speed of processing complex information compared to controls (Figure 2). None of the other cognitive measures or psychological variables assessed in the current study reached statistical significance following the intervention (see Table 2).

**Table 2:** Mean change-score comparisons between martial arts training and controls on outcome measures.

	**Martial arts**	**Controls**	***t***	***P***	**Cohen's *d***
	**(*n* = 20)**	**(*n* = 19)**			
	***M* (*SD)***	***M* (*SD)***			
Inhibition and shifting rate	−128.8 (139.3)	−3.34 (103.6)	3.18	0.003	1.02
Processing speed	−74.2 (60.2)	−11.8 (53.6)	3.41	0.002	1.09
Sustained attention	−0.009 (0.09)	0.02 (0.04)	1.07	0.29	0.37
Flexibility and problem Solving	−0.16 (3.48)	0.50 (3.68)	0.56	0.58	0.18
Self esteem	−0.22 (4.58)	0.45 (4.49)	0.47	0.64	0.15
Aggression	0.20 (2.57)	0.45 (5.28)	0.20	0.85	0.06

*Due to class scheduling conflicts, there was a reallocation or attrition of 8 pupils between the two groups during the second week of training, and 2 additional participants were discrepant for Ritalin between baseline and post-training testing, excluding their cognitive data from the final analyses. The final sample consisted of 20 participants in the martial arts training group and 19 in the control group*.

Based on our recent findings on hormonal reactivity during martial arts, we sought to examine whether initial OT and CT reactivity would predict outcome change scores in the group receiving martial arts training. OT and CT reactivity was indexed by subtracting baseline from peak-training values, as well as by AUC_G_ and AUC_I_. To this end, linear regression analyses were conducted, with OT and CT reactivity used as predictors and cognitive and psychological change scores as criterion variables. Model coefficients and fit are presented in Table 3.

**Table 3:** Standard multiple regression analyses for predicting response to cognitive and psychological variables from hormonal reactivity.

**Criterion and predictor variables**	**Unstandardized coeff**.		**Stand coeff**.		**Overall model fit**				
	***B***	***SE***	**β**	***p***	***R***	***R^**2**^***	***Adj. R^**2**^***	***F*_**(2, 20)**_**	***p***
**Inhibition and shifting rate**					0.15	0.02	−0.07	0.26	0.78
Oxytocin	−0.52	2.14	−0.06	0.81					
Cortisol	−0.04	0.07	−0.13	0.59					
**Processing speed**					0.72	0.52	0.47	11.2	<0.001
Oxytocin	−7.78	2.12	−0.60	0.001					
Cortisol	−0.11	0.07	−0.26	0.13					
**Sustained attention**					0.16	0.02	−0.07	0.26	0.77
Oxytocin	0.0001	0.002	0.01	0.95					
Cortisol	0.00004	0.00006	0.15	0.52					
**Flexibility and problem solving**					0.33	0.11	0.02	1.28	0.3
Oxytocin	−0.03	0.05	−0.14	0.53					
Cortisol	0.0001	0.0001	0.35	0.13					
**Self esteem**					0.55	0.30	0.24	4.55	0.02
Oxytocin	−0.002	0.002	−0.35	0.16					
Cortisol	0.00007	0.00002	0.69	0.007					
**Aggression**					0.45	0.20	0.13	2.68	0.09
Oxytocin	−0.09	0.04	−0.48	0.03					
Cortisol	0.001	0.001	0.22	0.31					

*Stand. Coeff., Standardized Coefficients; SE, Standard Errors*.

As can be seen in Table 3, the first regression model was significant (*p* < 0.001), explaining 47% of the adjusted variance. OT reactivity significantly predicted change in processing speed, such that higher hormonal reactivity was related to faster speed of processing (*p* = 0.001, BF_10_ = 89.9). In the second regression analysis, analyzing predictors of change in self-esteem, the overall regression model was significant (*p* = 0.02), explaining 30% of the adjusted variance. Overall CT response was found to be a significant predictor, such that higher response predicted increase in self-esteem (*p* = 0.007, BF_10_ = 3.43). Finally, the third regression approached accepted significance (*p* = 0.09), explaining 13% of the adjusted variance. OT reactivity was a significant predictor, such that greater OT response predicted greater reduction in aggression (*p* = 0.03, BF_10_ = 1.60). Models predicting changes in inhibition and shifting rate, processing speed, and flexibility and problem solving by OT and CT reactivity were not found to be significant (see Table 3).

## Discussion

The present exploratory study investigated the feasibility and efficacy of an extended martial arts training program which, differentiating from the recent single session studies (23, 24), integrated martial arts practice into the school schedule of at-risk youths. Compared to the sport-as-usual control group, a significant improvement was observed following the 6-month martial arts intervention on several measures of executive functions, including inhibition and shifting rate and speed of cognitive processing. Aggression and self-esteem remained unchanged in the experimental and control groups. Conversely, hormonal reactivity following the initial session of martial arts practice predicted reductions on measures of aggression and self-esteem, as well as an improvement in processing speed, following the intervention.

Martial arts were introduced to at-risk youths to assess whether changes witnessed in the few studies with low-risk youths, could be replicated in at-risk youths. The suggestion that martial arts may improve executive functions in normative youths has been quoted widely (16), although there is surprisingly little evidence backing up this finding. Introducing martial arts as an intervention with at-risk youths to improve executive functions showed promising, albeit preliminary, results. The current study was the first study to use computerized testing measures to test executive functions following martial arts training. Compared to a control group, the martial arts group improved significantly on key executive functions.

Our findings further reinforce a recent multi-site exercise program for children, which included martial arts (33) and a whole-school bi-weekly martial arts intervention (13). Both studies demonstrated significant improvements in participants' self-reported experiences of self-control following the intervention. It is recognized that sport interventions appear to enhance general cognitive abilities and in-class learning. However, programs that incorporate higher cognitive loads, target executive functions, and have some element of challenging group interaction appear to be the most effective at improving higher cognitive functions in the long-term (34). The results of the present study are consistent with these earlier studies, indicating that repetitive use and challenge in martial arts improve executive function performance even on tasks unrelated to martial arts practice.

The finding that inhibition, rather than flexibility or problem solving, is most enhanced by martial arts practice is in accordance with research into ADHD interventions, which consistently demonstrate inhibition as the most amenable executive function to training (18). It appears that inhibition, while being highly predictive of criminal risk (35), may also be most malleable with targeted interventions, as demonstrated by martial arts practice. Martial arts specifically challenge inhibitory skills in participants, with the character traits of restraint and self-control enhanced and refined during long-term martial arts practice (36). The current study is the first to demonstrate objective changes in inhibitory function through martial arts training.

The increase of self-control and inhibition during martial arts practice witnessed in this study may be potentially akin to the muscle strengthening effect seen in sports practice in general, such that the more the muscle is used, the more it improves (37). Namely, the more inhibition of behavior is trained, the greater it's responsivity even outside of the martial arts *dojo*. Furthermore, the observed improvement in speed of processing within the context of an inhibition challenging task, indicates that as this “muscle” is trained, it becomes faster as well as stronger. Our findings also reinforce the effect of prolonged training and translatability of behavioral self-control (as trained during the intervention) to cognitive inhibition computerized tasks (38). If, as Berkman et al. claim, behavioral, cognitive, and emotional inhibition all involve the right inferior frontal gyrus (rIFG), then this “muscle” training should make all neural processes involving the rIFG faster. Consistently, the present study found improvement in speed of processing selectively within the domain of inhibition, rather than other cognitive domains, such as sustained attention, which likely utilize other areas of the frontoparietal network (39).

Although the cognitive findings reinforced predictions based on previous research with normative youth, the current study found no significant differences in aggression or self-esteem levels following the intervention. This finding was unexpected given the previous positive findings, especially in the field of aggression, a finding which had been replicated in multiple populations and formed the basis for the current study (8). Reducing aggression in at-risk youth is particularly difficult in boys, and this difficulty is evident in the challenge of personalizing standard psychological treatments to specific populations (40), as well as optimizing treatments that show potential for benefitting these populations (41). Reducing aggression when at a greater risk of repeated exposure is thus extremely challenging, more so, it appears, than with low-risk youth. It seems then that the rapid decreases in aggression reported following martial arts practice in low-risk youth may be more difficult to achieve among high-risk youth, and so the regular practice identified by Basiaga-Pasternak et al. (42) may be crucial here to reducing aggression.

Another element of martial arts practice, in addition to regularity, may be reactivity to martial arts practice. The finding that OT reactivity in the initial session was related to greater subsequent decrease in aggression scores and faster reaction times following the intervention may suggest the possibility that a potential threshold may exist, which once crossed, cognitive and behavioral changes may occur. It should be noted that to date, the incongruity in findings on the effect of martial arts on aggressive behavior, with some studies reporting martial arts practice to increase aggression and others to decrease aggression (8), has been attributed to differences between traditional vs. modern (aggression inducing) martial arts styles. However, it is also possible that these differences in findings may to some extent be influenced by hormonal reactivity to the martial arts practice. Thus, although the findings of this small sample are limited, the current study introduces a new potential variable to the study of martial arts that may differentiate between those who derive benefit from martial arts practice in reducing aggression and those who do not, or could even experience an increase in aggression levels.

Consistently, OT responsivity to social interventions have been related to greater responsivity to the enhancement of well-being following group singing (43) and greater improvement in perspective taking following conflict resolution programming (44). It has been theorized that increased OT reactivity may indicate true reciprocal interactions, as opposed to superficial participation (45), although this has not yet been examined in intervention research. Although conflicting results predominate the field of aggressive and conflict related behaviors, they mostly relate to administered OT, rather than naturally produced OT in response to an intervention (46). It appears that OT reactivity might indicate the level of potential benefit experienced following a social intervention and thus enhance the intervention's potential effect. Additional research is required to extrapolate the exact role of OT in promoting reactivity to a social intervention, as well as on individual and group characteristics that promote OT reactivity, such as touch, aerobic exercise, and individual personality traits.

Finally, increased CT levels throughout the training were found to predict increased self-esteem following the intervention. Dampened CT levels have been associated with at-risk youths (47), as well as the desire to perform high-risk behaviors (48). Thus, it is possible that the relationship between increased CT and self-esteem is related to increased ability to enjoy the martial arts intervention. As demonstrated in previous research, the more one enjoys participation in the sport, the greater the effect of the sport on increasing self-esteem (49). With respect to the current study, this would suggest that the greater CT response predicted the propensity to enjoy martial arts training, possibly leading to greater impact on self-esteem although this link appears to be tenuous. Self-esteem during adolescence is notoriously volitile (28), and its volatility may be even greater in the current high-risk population. Another intervention study for youth with ADHD (50) reported similar findings, indicating no improvement in self-esteem while improving cognitive functions, which is consistent with the aformentioned suggestion. As with the OT findings, the relationship with CT levels may indicate a minimal threshhold for martial arts participation, although pending a larger scale study, this idea should be presently regarded as a working hypothesis.

The current study was designed as exploratory, with measures selected based on the few prior studies conducted in this area using different populations. For these reasons, before a larger randomized control study could be designed, the current study was performed to examine whether outcomes could be replicated in at-risk youths and whether they were influenced by physiological reactivity to the intervention. Importantly, the study was conducted in as close to real-life conditions as possible and designed not as a clinical intervention, but rather an intervention requiring little clinical or psychological training. Indeed, the ecological validity of the setting was a major strength of the current study. Unfortunately, this design also entailed a number of practical challenges. Primarily, a major weakness of the study was the lack of randomization. In order to retain the school-based location and work with unstable settings and systems, we had to relinquish our requirement of randomization in order to comply with school scheduling. Given that there was little data to reinforce the hypotheses of a supposed benefit of martial arts training with at-risk youth, this was considered a prerequisite for a future RCT. There were a number of other difficulties encountered with this volatile population. Whereas, martial arts as a chosen afterschool activity in a normative population typically has eager participants, the participants in the current study had poor discipline. Additionally, the teachers had difficulty reinforcing punctuality and attention, and several classes were shortened or canceled due to participants' poor behavior. The volatility of the establishments studied was reflected in schedule changes and school absences. Thus, attendance for the martial arts intervention proved to be an unsatisfactory measurement, as classes were interrupted with youths walking out, arguing with teachers or other students, or emotionally responding to the volatility in the wider school environment. Given the novelty of the intervention in this setting, these elements were not accounted for in the assessment constructs, and future research in such settings would require an observatory measure for actual youth's participation in the intervention. Furthermore, given the difficulty to enlist parents and teachers into the evaluation protocol, we had to rely only on self-report measures of self-esteem and aggression. Finally, the relatively small sample used in the present study requires future replications to attain a higher statistical power and to potentially generalize the present pilot results to larger populations with important implications to the literature of at-risk individuals.

Despite the outlined difficulties, a number of qualitative observations not captured by our measures should be acknowledged. There were youths who attended most sessions, built strong relationships with the instructors, and were active participants in conversations held during the group on the use of skills and inhibition of detrimental urges outside of the group setting. These discussions demonstrated possible change in participant schemas and commitments, which could not be fully captured in questionnaire recordings. Future research should make an effort to assess these qualitative aspects to more fully capture these potentially meaningful effects. Adding such assessments would help capture both parental and youth experiences and potentially elucidate pathways of psychological change.

Bearing in mind the significant limitations of the current study coupled with the promising findings, which will hopefully pave the way for future research, the current study reinforces the suggestion that martial arts are a positive intervention for at-risk youth. Empirical studies and day-to-day practice with difficult populations often demonstrate wide variations in findings and, in turn, the treatments and interventions provided to similar populations vary widely (51). On the one hand, researchers attempt to dispel the common belief that nothing works with youth with severe behavioral problems (41), but on the other hand, supplementary interventions, such as martial arts, are touted as game changers (e.g., in the field of executive functions), with very little evidence to back up such claims (16). The current study has cautiously demonstrated that martial arts is indeed beneficial to at-risk youth and justifies its continued inclusion in a range of settings for both at-risk and normative youth. Furthermore, this study, together with the recent findings indicating the added benefit of martial arts in increasing OT for at-risk youth (24), suggest that greater hormonal increases may also indicate greater potential for behavioral and psychological benefits from this practice.

In conclusion, this study was the first to use objective neuropsychological measures of executive functions, demonstrating the potential utility of martial arts training in improving some cognitive functions, such as processing speed and inhibitory behaviors among at-risk youths. Despite the challenges that many alternative educational establishments experience on a day-to-day basis, the intervention was administered within the educational framework for which it was designed, thus enhancing its ecological validity. The current study further demonstrated the potential importance of hormonal reactivity as a mediating factor when measuring martial arts intervention efficacy. Given the dearth of rigorous and systematic research in the field of martial arts, we have described its feasibility and highlighted a number of inherent challenges, while pointing to potential findings, in the hope that this pioneering work would serve an impetuous for additional research in this area. Future research is needed to further validate and generalize the present findings in larger samples and different populations. Thus, awaiting further replications, this enjoyable, readily available, group intervention could potentially be used to improve cognitive and psychological functions in at-risk youths and, in turn, make a positive impact on their lives and the lives of their families.

## Data Availability Statement

The raw data supporting the conclusions of this article will be made available by the authors, without undue reservation.

## Ethics Statement

The studies involving human participants were reviewed and approved by the research was approved by the Institutional Review Board at Bar-Ilan University, the Israel Ministry of Education Ethics committee, and the Helsinki Ethics Committee of Hadassah Hospital in Jerusalem, as required by the Ministry of Health. Written informed consent to participate in this study was provided by the participants' legal guardian/next of kin.

## Author Contributions

AH-G conducted the study and assisted with study conceptualization, data interpretation, and wrote the first draft of the manuscript. BL assisted with data collection and interpretation and manuscript preparation. RF assisted with study conceptualization, data interpretation, and manuscript preparation. OZ-S assisted with data interpretation and manuscript preparation. YR conceptualized the study and assisted with data analyses, interpretation, and manuscript preparation. All authors contributed to the article and approved the submitted version.

## Funding

This research was supported by the Ministry of Science, Technology & Space, Israel (Grant #3-13631) to YR and RF.

## Conflict of Interest

The authors declare that the research was conducted in the absence of any commercial or financial relationships that could be construed as a potential conflict of interest.

## Publisher's Note

All claims expressed in this article are solely those of the authors and do not necessarily represent those of their affiliated organizations, or those of the publisher, the editors and the reviewers. Any product that may be evaluated in this article, or claim that may be made by its manufacturer, is not guaranteed or endorsed by the publisher.
